# Telecoaching and Migraine: Digital Approach to Physical Activity in Migraine Management. A Scoping Review

**DOI:** 10.3390/jcm14030861

**Published:** 2025-01-28

**Authors:** Ignazio Leale, Vincenzo Di Stefano, Angelo Torrente, Paolo Alonge, Roberto Monastero, Michele Roccella, Filippo Brighina, Valerio Giustino, Giuseppe Battaglia

**Affiliations:** 1 Sport and Exercise Research Unit, Department of Psychology, Educational Sciences and Human Movement, University of Palermo, 90144 Palermo, Italy; valerio.giustino@unipa.it (V.G.); giuseppe.battaglia@unipa.it (G.B.); 2 Department of Psychology, Educational Science and Human Movement, University of Palermo, 90129 Palermo, Italy; michele.roccella@unipa.it; 3 Department of Biomedicine, Neuroscience, and Advanced Diagnostics (BiND), University of Palermo, 90129 Palermo, Italy; vincenzo.distefano07@unipa.it (V.D.S.); angelo.torrente@unipa.it (A.T.); paolo.alonge01@unipa.it (P.A.); roberto.monastero@unipa.it (R.M.); filippo.brighina@unipa.it (F.B.)

**Keywords:** telecoaching, migraine, adapted physical activity, exercise, digital tools, COVID-19, lifestyle changes

## Abstract

Migraine is a common neurological disorder, affecting approximately 15% of the European population and is among the main causes of years lived with disability. In the context of increasing digitalisation, telecoaching (TC) is a new training modality that involves the use of digital tools to access and manage training services remotely. Given the well-documented benefits of physical activity in migraine management and the rapid expansion of digital health services following the COVID-19 pandemic, this scoping review aims to evaluate the use and feasibility of TC-based training programs in individuals with migraine. A systematic search was conducted on multiple databases (PubMed, Web of Science, and Scopus) identifying 1507 studies, of which only 3 met the inclusion criteria. These studies collectively involved 181 participants with migraine and assessed various training programs, including aerobic training, resistance training, and physical therapy. Most training programs showed statistically significant improvements in several variables, including severity, duration, and frequency of migraine attacks. However, based on our study, there is limited evidence to suggest that TC training is beneficial for migraine patients. These findings underscore the need for further investigation, with more rigorous methodologies, higher-quality trials, and larger sample sizes to better establish the efficacy of TC training as a preventive and therapeutic approach for migraine.

## 1. Introduction

### 1.1. Incidence and Impact of Migraine

Migraine is a highly common neurological disorder that affects approximately 15% of the European population [1], resulting in the second most severe disorder in terms of years lived with disability [2], and is the main cause of disability in the 15–49 age group [2]. It is a cyclic disorder characterised by recurrent attacks of moderate-to-severe headaches that may be pulsating in quality and unilateral, accompanied by nausea and intolerance to light or noise [3]. Moreover, one of the characteristics is that the pain usually worsens with physical exercise [3]. Some patients also experience transient focal neurological symptoms (usually lasting up to one hour each) before or during an attack, called aura, which configure the migraine with aura subtype [3].

Migraine creates a debilitating condition that causes work absenteeism or presenteeism (i.e., working with limited efficiency) and reduced participation in social activities [4]. Some consequences are high economic losses [5] and an increased risk of side effects due to overuse or misuse of medications [6].

### 1.2. Pathogenesis and Management of Migraine

In recent decades, much progress has been made in understanding the physiopathology, genetics, and neurophysiology of migraine. There is strong evidence that migraine may depend on alterations in cortical excitability (i.e., increased), which may lead to maladaptive patterns of brain plasticity, especially when the frequency of headache attacks increases (i.e., transformation from episodic to chronic migraine) [7,8,9].

The progress allowed the creation of new opportunities for the diagnosis and management of this disorder, even in rare familial forms [10,11]. There are two different approaches, which include the use of acute medication to stop a migraine attack, or preventive medication to reduce the frequency, duration, and severity of the pain episodes. The guidelines recommend the use of preventatives in case of debilitating headaches occurring for 2 or more days per month [12]. However, traditional oral prophylaxes may not be well tolerated or accepted by patients due to the possible side effects [13,14].

### 1.3. Non-Pharmacological Treatment of Migraine

For the above-mentioned reasons, non-pharmacological treatments, such as self-management strategies, manual therapy, physical activity, or physical exercise progressively gained interest as valid alternatives to conventional therapies [15,16,17]. Sport has a controversial role in migraine [18,19]. Although sport has been associated with a worsening of migraine attacks [20,21], there is evidence that regular physical activity or physical exercise could play a primary role in preventing them [22,23]. This phenomenon could be related to the intensity of exercise. High-intensity exercise (mainly present in sport) is a migraine trigger [20,21], while regular and moderate-intensity exercise (mainly present in physical activity) plays a role in attacks prevention [19,23]. Busch and colleagues [23], in their review, showed that regular, planned, and structured physical activity was associated with several benefits in this target population. The beneficial effects of exercise on migraine may be related to better pain modulation. Indeed, several studies demonstrate the analgesic effect of aerobic exercise [24,25]. Despite these findings, it has been shown that patients with migraine are more sedentary than healthy subjects [26]; a sedentary lifestyle that has further increased due to the recent COVID-19 pandemic, in several neurological disorders [27,28]. On the other side, the recent COVID-19 pandemic raised the need and gave impulse for the development of telemedicine.

### 1.4. Aerobic and Resistance Training: Evidence-Based Benefits

Recent literature demonstrated that exercise plays a significant role in the management of migraine. Specifically, aerobic activity has been shown to be particularly effective in reducing both the frequency and intensity of headache episodes, as well as enhancing the overall well-being of patients [29,30]. Indeed, as shown by the current literature, aerobic activity improves not only the specific parameters of migraine (frequency, intensity, and duration), but also the cardiovascular function, the affective discomfort [30], and the quality of life (QoL) of these patients [29]. A recent systematic review highlighted that moderate aerobic training is the most effective approach for reducing migraine-related disability [31]. Furthermore, the findings suggest that combining pharmacological treatment with aerobic training is more effective than drug treatment alone in decreasing both the frequency and intensity of migraine attacks [31].

Resistance training has been shown to positively contribute to migraine management by enhancing overall muscle strength and physical resilience, as well as reducing the frequency of headache attacks [32]. Muscle strengthening and reconditioning, particularly focused on the neck, shoulder, and upper limb muscles, could be the mechanisms responsible for the therapeutic effects of resistance training [32]. Furthermore, resistance training facilitates the increase and preservation of lean muscle mass, which can help reduce sarcopenia [33]. Research indicates that an increase in lean muscle mass is associated with a reduction in the frequency of migraine episodes [34]. These findings highlight the importance of incorporating resistance training into a comprehensive approach to migraine management.

### 1.5. Telecoaching: New Training Approach

A recent study showed the important association between migraine and some neurodegenerative disorders. For example, patients with migraine are more likely to develop Parkinson’s disease (PD) than subjects without migraine [35]. Kim and colleagues also found a relevant association between migraine and Alzheimer’s disease [36]. The authors demonstrated that individuals with a history of migraine had a higher prevalence of Alzheimer’s disease. The association was even stronger in young and obese subjects with migraine [36]. These findings suggest that migraine may be a risk factor for some neurodegenerative diseases, so proper migraine management (as well as an active lifestyle) might help reduce this risk. For these reasons, there is a need to develop new intervention strategies to contrast migraine and increase physical activity levels. This approach could also induce changes in brain plasticity that could have a beneficial effect on headache frequency.

A new training modality could be represented by telecoaching (TC). Unlike traditional training, where exercises are performed in specific facilities and coach–athlete communication occurs in person, TC utilises technological and digital tools, such as computers and mobile devices, to deliver and manage training services remotely. This new approach of distance communication offers greater flexibility and facilitates access to the training. Technological tools allow the trainer to send training materials and monitor adherence to the program, while allowing patients to train independently, overcoming obstacles such as time, distances, and transport [37]. This new approach could serve as a primary strategy to increase adherence and engagement of individuals, reducing sedentary lifestyles. The effectiveness of TC in other populations and diseases has already been demonstrated, including its efficacy in the elderly population [38,39], in respiratory diseases [40,41], in patients with metabolic or cardiac diseases [42,43], as well as in patients with other neurological diseases such as Charcot–Marie–Tooth [44,45]. Therefore, TC could be one of the main tools to be used to increase physical activity levels in migraine patients, reducing their sedentary lifestyle and promoting improved QoL, autonomy, and self-esteem [46]. However, potential issues related to internet connection, as well as video and audio quality, should be considered to ensure the effectiveness of this modality [47]. TC aligns well with telemedicine and eHealth, which, despite its various approaches and challenges primarily related to the complexities of app development [48], has been extensively utilised and studied [47], as well as with the use of wearable devices for the benefit of human health [49]. While some patients express a preference for a hybrid care model [48], telemedicine consultations demonstrated a quality of care comparable to that of traditional headache outpatient consultations, offering a more cost-effective solution for patients [47], with also a positive endorsement from neurologists [50]. However, the use of telemedicine is significantly influenced by geographic location [51]. Countries such as the United States, China, and Norway, which benefit from advanced technology, make extensive use of telemedicine [52,53]. In contrast, countries such as Lithuania have not adopted this tool to the same extent. A recent study indicated that only 17% of migraine patients in Lithuania received remote consultations, compared to 57.5% of patients in the United States [51,52]. Positive experiences with telemedicine may encourage wider adoption of this approach.

### 1.6. Aim of the Study

Considering these premises, this scoping review aims to identify the available evidence on the use and feasibility of TC training programs in migraine patients.

## 2. Materials and Methods

This scoping review was developed according to the PRISMA guidelines for systematic reviews and meta-analysis [54]. The protocol was not recorded in a specific database but was developed before the study.

### 2.1. Eligibility Criteria

Inclusion criteria were: (a) original research with full text written in English; (b) all study designs different than reviews, meta-analyses, letters to editors, and theses; (c) studies with training programs performed in TC for migraine patients; (d) studies published over the last decade, concluding in September 2024. No gender difference between males and females was used as an exclusion criterion. The population, intervention, comparison, outcome, and study design (PICOS) framework was used as shown in Table 1.

### 2.2. Data Collection

Major databases, including PubMed (NLM), Web of Science (TS), and Scopus, were used to find useful articles for this study. Keywords included: exercise, physical activity, telecoaching, migraine, and headache. The different terms have been divided into 3 groups. In Group A, the terms “migraine” and “headache” were entered; in Group B, the terms “exercise” and “physical activity”; and in Group C, the term “telecoaching” was entered. The Boolean operators ‘AND’ and ‘OR’ were used to analyse the three categories. Matching examples were “migraine” AND “physical activity” AND “telecoaching”. All items found were transferred to Endnote software for the analyses (Version X20 for Windows 11, Thomson Reuters, New York, NY, USA).

#### 2.2.1. Study Selection

**Figure 1:**
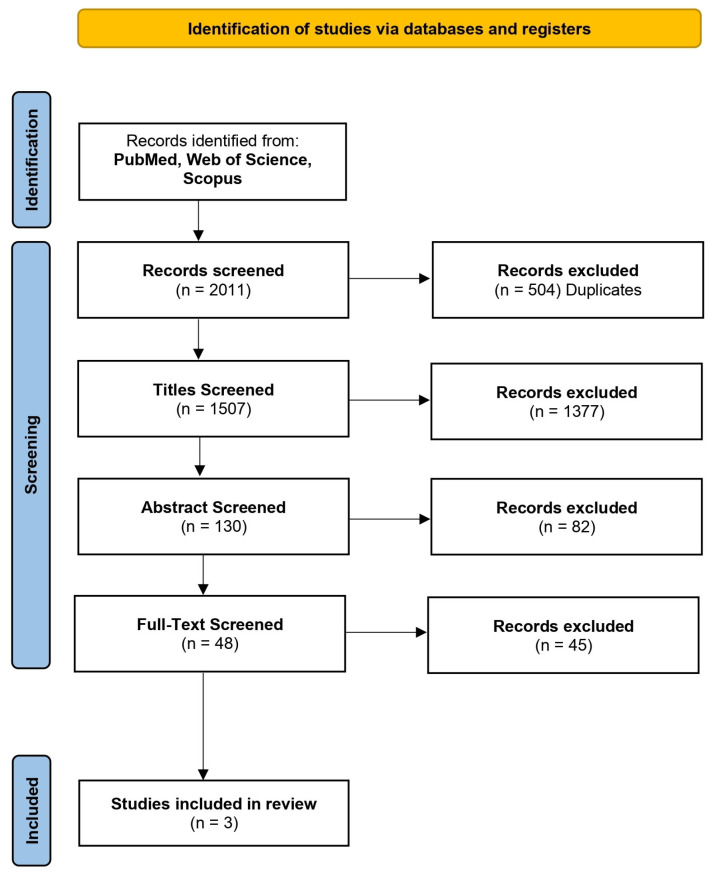
The flow diagram representing the selection process of records.

Database analysis, identification, and elimination of duplicates were carried out by a single researcher. Subsequently, two authors independently analysed the studies (I.L.; V.D.S.). In detail, in the initial phase, the title and abstract of each study was examined; in the final phase, the researchers double-checked the entire text to confirm that the selected studies met the inclusion and exclusion criteria. In case of disagreements between the two raters on the inclusion or exclusion of a study, a third researcher was consulted (G.B). A Microsoft Excel spreadsheet (Microsoft Corp, Redmond, WA, USA) was used to report the study information including year of publication, age of sample, gender, aim of the study, results, and TC training protocol. The PRISMA flow diagram (Figure 1) illustrates the process by which the articles were selected.

#### 2.2.2. Quality and Risk of Bias Assessment

Two authors (I.L.; V.D.S.) evaluated the quality and BIAS of all the studies included, using a modified version of the “Downs and Black Checklist” [55]. In the modified version of the Downs and Black checklist, the score of item 27 (concerning the power of the study) was changed from 0–5 to 0–1, so that a study would receive a score of 0 in case the statistical power was below 80%, and a score of 1 if the statistical power was above 80% [56]. Taking this into account, the final checklist score changed from 32 to 28. The quality of the studies was divided into four levels: excellent (26–28), good (20–25), fair (15–19), and poor (<14) [57,58]. Another researcher (G.B.) compared the authors’ results for each study and discrepancies were resolved through a consensus meeting.

## 3. Results

### 3.1. Study Identification

A total of 2011 studies were identified through electronic databases. In total, 504 studies were eliminated because they were duplicates; 1507 titles and 130 abstracts were analysed. The full text of 48 studies was analysed; of these, only 3 studies agreed with the inclusion and exclusion criteria. Specifically, the three included studies analysed the efficacy of a TC aerobic training program [59], a TC resistance training program [60], and a TC physical therapy [61]. A total of 181 participants with migraine were included in this review. All included studies presented data on both males and females, but female sex was prevalent (n = 139, 76.7%). More detailed information about the study selection process and the characteristics of the included studies can be found in Figure 1 and Table 2.

### 3.2. Methodological Quality

For each study, the methodological quality was analysed through the modified version of the Downs and Black Checklist. All studies included were rated as “fair quality” [59,60,61]. These results must consider that some items on the checklist are difficult to use in sport and exercise field. For example, the use of a double-blind study design is difficult due to the presence of a program with physical activity.

### 3.3. Aerobic Training

Among the identified studies that used aerobic training, there is the article by Santiago and colleagues [59]. Researchers evaluated the benefits induced by the association between exercise and medication compared to medication alone in patients with migraine. The inclusion criteria for this study were a diagnosis of chronic migraine, normal cardiac and neurological examination, and being sedentary for at least 3 months. Based on these criteria, 60 patients were included and divided into two groups: a control group receiving pharmacological treatment only, and an experimental group combining pharmacological treatment with a TC training program. The control group was treated with amitriptyline (25 mg/day) for 12 weeks. The experimental group followed the same pharmacological treatment but integrated with an aerobic training program. The aerobic training program, performed via TC, consisted of free outdoor walking of 40 min with a weekly frequency of 3 times for 12 consecutive weeks. TC strategies included weekly phone calls to monitor progress and motivate participants, the flexibility for participants to choose when and where to perform the exercises, a spreadsheet with a detailed training plan to ensure the correct execution of the exercises, and a supervised training session with a movement expert. The following parameters were evaluated: frequency (days/month), intensity, and duration/day of migraine attacks (6 h, 12 h, 18 h, and 24 h), body mass index, Back Depression Inventory, and Beck Anxiety Inventory at baseline and the end of the third month. Both groups showed a decrease in the frequency, intensity, and duration of migraine episodes. However, the efficacy of amitriptyline increased when combined with the TC training program.

### 3.4. Resistance Training

Of the identified studies, Madsen and colleagues [60] used a TC approach to examine the effectiveness of a resistance training program and a postural training program on the frequency and duration of migraine. A total of 60 subjects were randomised into two groups: the control group and the experimental group. The control group was instructed on ergonomic and postural correction and performed postural exercises three times a day for 10 weeks. The experimental group performed a resistance training program, with three sessions per week for 10 weeks. The resistance training program involved the execution of specific shoulder exercises with progressive intensity: initially 70%, and later 80% of 1RM. All exercises were performed with elastic bands. TC approach was applied in both groups and the strategies included a free choice of when and where to perform the exercises, weekly calls to monitor exercise adherence and manage progress and motivate subjects to protocol adherence, and some supervised sessions to manage the correct execution of exercises. In addition, participants were given a migraine diary to record the frequency, duration, and intensity of headache episodes. Although no statistically significant changes were detected between groups, the experimental group reduced the frequency of headache episodes by 11% and the duration by 10%, while the control group showed a 24% reduction in frequency and 27% in duration 27% of headache attacks.

### 3.5. Physical Therapy

Mehta and colleagues [61] used a randomised controlled trial to evaluate and compare the effectiveness of physical therapy (stretching) and yoga therapies, applied with a TC approach, for the adjuvant therapy of standard pharmacologic treatment in patients with migraine. Subjects diagnosed with migraine, over 18 years old, and with at least five headache attacks per month were included in the study. A total of 61 patients were distributed in three groups: physical therapy, yoga therapy, and standard therapy. The physical therapy group practiced relaxation exercises, stretching of the neck muscles, and cardiorespiratory endurance training (30 min of free walking). The yoga therapy group performed several specific exercises, including the position of the butterfly (Bhadrasana), the position of the cobra (Bhunjagasana), and the touch of the feet standing (Padhastasana). The standard treatment group continued the usual medication treatment without any additional physical therapy. Participants performed the specific program for 12 weeks. All subjects received lifestyle advice including obtaining adequate rest, not skipping meals, avoiding bright lights, and using a headache diary to identify headache triggers. TC strategies, both physical therapy and yoga groups, included free choice of when and where to perform the exercises, constant weekly calls to monitor the progress, encouragement for participants, and motivation for them to perform the recommended intervention regularly. All groups showed statistically significant improvements in the frequency and severity of headaches. Additionally, pain assessments revealed improvements in all groups compared to baseline.

Table 3 summarises the different types of treatment and the training programs applied to the TC approach.

## 4. Discussion

The primary aim of this scoping review was to investigate the use and feasibility of TC training programs in patients with migraine. The main outcomes assessed included the intensity, frequency, and duration of headache episodes, as well as pain perception and any potential adverse events. The present study showed very interesting results. First, this scoping review comprised more than 60% of women, a distribution that aligns with numerous epidemiological studies reporting a female to male ratio of 3:1 [62]. Second, the review demonstrated that in the analysed studies, an exercise conducted in TC significantly reduced the burden of headache attacks, leading to decreases in their intensity, frequency, and duration.

### 4.1. The Benefits of Physical Exercise in Migraine Management

The relationship between exercise and migraine is very complex [63]. Many studies show how physical exercise may represent a trigger for migraine attacks [18,64]. Nevertheless, for many authors, these negative aspects would depend on the excessive intensity of exercise [20] or performing an inadequate warm-up [65]. We can hypothesise that it may depend on the rise of serum calcitonin gene-related peptide (CGRP) levels occurring during exercise for its cardiovascular role in vasodilation [66,67] in a susceptible subject. In contrast, many studies highlight the positive effects of exercise in this population. Indeed, exercise would seem to significantly reduce the impact of migraine, improving the lifestyle and QoL [68,69]. Moreover, the absence of exercise, which increased during the COVID-19 pandemic and related social restrictions, has been shown to affect sleep quality in migraineurs [70]. The benefits of physical exercise would seem to depend on an increase in the plasma level of β-endorphins [71]; in fact, the β-endorphin level appears to be lower in migraineurs than in healthy subjects [72]. There is evidence that exercise increases the concentration of β-endorphins in healthy individuals [73,74]. Köseoglu and colleagues [75] showed a similar effect on migraine patients both after a single training session and after a prolonged training program. Further studies in the literature highlight additional benefits of exercise in this population, including increased cardiovascular and cerebrovascular capacity, as well as improvements in psychological states such as depression, stress, and anxiety [63,76]. Moreover, exercise has been shown to improve pain perception [77]. After exercise, patients show a reduction in pain-related fear and its perception [77]. In addition, migraine is a risk factor for excessive drug intake [78]. Overuse of medications in migraine patients causes increased disability, depression, anxiety, and fear, as well as increased pain and headache frequency (i.e., medication overuse headache) [79]. Consequently, exercise can be proposed as a useful tool to counteract the high use of drugs. Despite these positive aspects, as shown by Lemmens and colleagues [80], the drop-out rate to exercise is very high in migraineurs, due to the lack of time to perform supervised exercise training sessions/week [80].

### 4.2. Telecoaching: A Digital Approach to Migraine Management

Considering the spread and convenience of telemedicine, the new technological solutions in managing migraine [81], along with the need for structural changes in healthcare [82], TC could represent a valuable approach for this population. By providing personalised guidance and remote support, TC aligns with modern healthcare trends and has the potential to enhance adherence to physical activity programs. This approach could be particularly beneficial for migraine patients, offering accessible and flexible training options that remove common barriers to exercise. However, it is important to acknowledge the psychological variables and the strong association between anxiety, mood disorders, depression, and migraine [83]. Participants’ attitudes toward TC may have played a crucial role in their performance in the included studies [82]. Positive attitudes toward this training modality can improve participant engagement, adherence, and overall satisfaction, while negative perceptions may reduce participation and limit the effectiveness of the intervention. This highlights the need to design user-friendly tools and methods that support acceptance of the condition and provide adaptive content and functionality [82]. However, attitudes toward telemedicine and eHealth are rarely reported or analysed as factors influencing participant performance in existing studies [84]. Future research should address this gap by incorporating these attitudes as a variable of interest, both in study design and analysis. To reduce stress, a major trigger of migraine attacks, Varkey and colleagues proposed the implementation of home training programs [85], incorporating TC training modality to enhance physical activity levels. Consistent with Varkey’s study [85], the articles included in this review have shown several benefits. Indeed, in every study included in this review, the dropout rate in the TC group did not exceed 30%, with one study that showed a 0% dropout rate for the TC training program [61]. This demonstrates how TC is a sustainable and accepted training methodology in this population. This finding is significant for patients who intend to perform a training protocol and for physicians who intend to advise active participation in a physical activity program for migraine patients. The sustainability of TC and the relative benefits (i.e., training in the preferred place, during the leisure time, breaking down barriers such as costs and structures [37]) make TC one of the main solutions for physical activity intervention. The sustainability of TC in migraine is in line with the scientific literature for other neurological diseases. Burns and colleagues evaluated the effectiveness of a TC resistance training program for the weakness of the dorsiflexion of the foot in children with Charcot–Marie–Tooth polyneuropathy. The intervention program attenuated the progression of weakness in dorsiflexion without side effects and with a low abandonment rate (8%) [44]. Van der Kolk and colleagues evaluated the efficacy of aerobic exercise performed in TC to relieve motor symptoms in patients with mild-severity PD. The reduction in motor symptoms demonstrated in the intervention group showed how TC can be a useful additional tool to manage this disease. Ninety-four percent of the participants completed the study, demonstrating high tolerance to the TC training modality. In agreement with Varkey and colleagues, our study suggests the possible implementation of TC in patients with migraine; although, the few included studies reported in this scoping review do not allow us to evaluate in detail the relationship between TC and migraine in detail. Future studies should analyse and evaluate the efficacy of a fully executed TC training program, using uniform outcome measures for migraine as recommended by the International Headache Society [86], with a randomised controlled trial design based on the guidelines of the American College of Sports Medicine (3–5 days per week, 20–60 min, with an intensity equal to 55/65–90% of maximum heart rate).

### 4.3. Strength and Limitations

The following limitations were identified in this study: firstly, the few studies included and the heterogeneity of the physical activity programs used could alter the generalisation of results, not allowing the correct relationship between migraine and TC to be assessed. Secondly, the quality of the studies was not excellent, a condition that could depend on the researchers’ lack of double-blinding (a condition that is difficult to apply with an exercise program to be performed). Additionally, the lack of homogeneity in the evaluation methods did not allow us to conduct a meta-analysis. Finally, the absence of a direct comparison between groups treated with and without TC represents a significant limitation of this review, as it does not allow us to assess in detail the true effects of TC. Thus, the observed benefits may not be exclusively attributed to TC.

Future studies should aim to design randomised controlled trials to clarify these effects and to evaluate the participant’s attitudes towards the TC, especially considering the absence of psychological component evaluations in the included studies. Despite these limitations, this review has several strengths. It highlights that TC is potentially safe, effective, and risk-free for migraine patients. An additional strength is the dropout rate: no included study showed a dropout exceeding 30% and one study noted a 0% dropout rate. Finally, to our knowledge, this is the first review in the literature to examine the application of TC in migraine patients, as well as the first attempt to evaluate novel treatments for the management of this disease and the exacerbation of symptoms observed during the COVID-19 pandemic [87].

## 5. Conclusions

The results of this review, based on GRADE guidelines, show low evidence that exercise performed in TC is beneficial in patients with migraine. Further studies are needed to show the efficacy, benefits, and safety of this training modality, as well as to establish guidelines for administering a TC training program in this population. Future research should focus on high-quality randomised controlled trials to clarify and isolate the effects of TC on migraine outcomes. These studies should aim to standardise physical activity programs and standardised outcome measures to enable more comparisons. Additionally, it is essential to assess participants’ attitudes toward TC in future studies, as this factor could significantly influence their performance and engagement. Addressing these gaps will help to clarify the role of TC as a treatment modality for migraine and establish evidence-based guidelines for its use.

## Figures and Tables

**Table 1:** The PICOS framework.

PICOS Components	Details
Population	Individuals with migraine.
Intervention	Telecoaching training program (aerobic training, resistance training, stretching, and physical therapy).
Comparison	The post-exercise migraine endpoints were compared to pre-exercise migraine endpoints within each study.
Outcome	Migraine endpoints (migraine days, attack frequency, pain intensity, and duration of migraine attacks).
Study design	Original articles.

**Table 2:** The aim of the studies and relative characteristics of the samples.

First Author, Year	Participants [F%], Age ± SD	Aim	Telecoaching Strategies	Downs and Black Score
Santiago, 2014 [59]	60 [88%], 31 ± 9	To compare the preventive treatment benefits of amitriptyline and aerobic training in patients with migraine.	- Weekly telephone calls to assess the progress of training. - One supervised training session. - Explanatory leaflet about the warm-up exercises.	18
Madsen, 2018 [60]	60 [68%], 32 ± Na	To examine the effectiveness of resistance training and postural exercise on tension-type headache frequency and duration.	- Weekly telephone calls to assess the progress of training. - Some supervised training sessions. - An exercise diary to monitor adherence and migraine intensity.	19
Mehta, 2021 [61]	61 [74%] 39 ± 8.24	To evaluate and compare the effectiveness of physical and yoga therapies in patients with migraine.	- Weekly telephone calls to assess the progress of training. - Some supervised training sessions. - An exercise diary to monitor adherence.	19

F: female; SD: standard deviation; and Na: not available.

**Table 3:** Types of program in telecoaching.

Author, Years	Exercise	Intervention (n)	Program (time)	Training Program	Main Results
Santiago, 2014 [59]	AT	TCG (30) vs. CG (30)	12 weeks of training, 3 times/W	TCG: 40’ free outdoor walk. CG: usual daily activities + drug treatment.	The drug was an effective treatment for chronic migraine, but its efficacy was increased when combined with AT.
Madsen, 2018 [60]	RT	TCG_1_ (30) vs. TCG_2_ (30)	10 weeks of training, 3 times/W	TCG_1_: shoulder exercises with resistance from the elastic bands. TCG_2_: ergonomic and posture correction + specific exercise for lumbar lordosis.	Both groups showed a reduction in the frequency and duration of migraine episodes.
Mehta, 2021 [61]	PT	TCG_1_ (20) vs. TCG_2_ (20) vs. CG (21)	12 weeks of training, 4 times/W	TCG_1_: progressive muscle relaxation exercise, self-stretching of neck muscles, isometric exercise of neck muscles, and cardiorespiratory endurance training (30’ of free walking). TCG_2_: Pranayama, and asana followed by Savasana. CG: usual daily activities.	Physical therapy and yoga, added to regular care, improved QoL and reduced the frequency of migraine.

TCG: telecoaching group; CG: control group; W: week; QoL: quality of life; AT: aerobic training; RT: resistance training; and PT: physical therapy.
